# Scheme of Development

**Published:** 1919-04-26

**Authors:** 


### UNIVERSITY COLLEGE HOSPITAL.

### Scheme of Development.

At the annual meeting of University College Hospital indications were given of developments which the authorities have either accomplished or are in hopes of bringing to fruition in the near future. We are now in a position to give details of the added responsibilities and the direction of future policy by which it is hoped to keep University College Hospital abreast of the times and the enterprise of other great medical schools. Briefly put the plans include:

Amalgamation with the Royal Ear Hospital, Soho.

Appointment of whole-time teachers of medicine and surgery, with whole-time assistants.

Establishment of a centre for the treatment of psycho-neurosis.

### The Royal Ear Hospital.

The Committees of University College Hospital and the Royal Ear Hospital, Dean Street, Soho, have agreed to amalgamate the institutions. They will be merged on the lines followed in the amalgamation of the National Dental Hospital and the University College Hospital four years ago. The Royal Ear Hospital will become a special department of the University College Hospital for ear, nose, and throat cases. The department will have twenty-four beds at its disposal for special cases. According to present plans, the Royal Ear Hospital will be reserved for the treatment of in-patients; the work attached to out-patients will be continued at the University College Hospital by means of daily clinics. The special department at the ear hospital will be managed by a sub-committee; otherwise the incorporation with University College Hospital is without reservation. If in the future it is thought desirable, the Committee has power to dispose of the building in Dean Street, Soho, for the purpose of bringing the new department nearer to University College Hospital. It may be mentioned that the Royal Ear Hospital was established in 1816. The hospital was rebuilt in 1904.

### Whole-time Teachers.

Perhaps a still more important step is the decision of the General Committee to approve a proposal to appoint whole-time teachers of medicine and surgery. The idea at present is to have one whole-time teacher in each branch. Each teacher will have two whole-time assistants. An adequate number of beds will be placed at their disposal. The hope of the Committee is that the Board of Education will contribute the funds necessary to pay the salaries of these teachers and their assistants. This scheme commends itself to the Committee as tending to help the teaching of medicine and surgery in the hospital, and as being in accordance with the policy of the Board of Education. Women students have been admitted to University College Hospital since October 1 last, and the arrangement is working to the satisfaction of all concerned.

### Nerve Diseases.

A further scheme, which is now under consideration, is the establishment, in connection with the hospital, of a centre for psycho-neurosis. This would be under the direction of Dr. Bernard Hart. In view of the nervous and mental disorders produced by shell-shock, and otherwise by causes incidental to the modern conditions of life, it is regarded as important that London should be provided with such a centre. If the scheme is embarked upon, it will probably be necessary to obtain suitable accommodation in the neighbourhood of the hospital.

